# Prospective comparison of the novel visual prostate symptom score (VPSS) versus the international prostate symptom score (IPSS), and assessment of patient pain perception with regard to transrectal ultrasound guided prostate biopsy

**DOI:** 10.1590/S1677-5538.IBJU.2018.0496

**Published:** 2019

**Authors:** M. Els, C. Heyns, A. van der Merwe, A. Zarrabi

**Affiliations:** 1 Department of Urology, Stellenbosch University and Tygerberg Hospital, Cape Town, South Africa

**Keywords:** Pain Perception, Lower Urinary Tract Symptoms, Literacy

## Abstract

**Objective:**

To compare the International Prostate Symptom Score (IPSS) and novel Visual Prostate Symptom Score (VPSS) in patients with lower urinary tract symptoms (LUTS), to correlate scores with uroflowmetry and prostate volume and assess patient perceptions regarding pain prior to, and after prostate biopsy.

**Materials and Methods:**

Patients with LUTS who had an indication for transrectal ultrasound (TRUS) biopsy were included. Patients completed the IPSS-, VPSS- and prostate biopsy pain assessment questionnaires. Assessment included uroflowmetry, post- void residual volume and prostate volume (measured with TRUS).

**Results:**

One hundred men were included. There were statistically significant correlations between the VPSS score and IPSS score (correlation coefficient (r) = 0.802); VPSS and Qmax (r = −0.311); VPSS and. Qave (r = −0.344); prostate volume with VPSS (r = 0.194) and Qmax (r = −0.260). The VPSS was quicker to complete than the IPSS (mean 100 vs. 165 seconds). The mean anticipated pain score before biopsy was 2.8 (range 0-6), and after biopsy (experienced pain) it was 1.8 (range 0-5). The pain during biopsy was less than expected in 67% of patients.

**Conclusion:**

In men with LUTS scheduled to undergo prostate biopsy, the VPSS score correlated positively with the IPSS score. Men with limited education take less time to complete the VPSS. Patient's perception of expected pain or discomfort during TRUS-guided prostate biopsy was significantly higher than the pain actually experienced during biopsy. Men with lower education level had significantly higher expectation of pain prior to biopsy, but similar pain during biopsy.

## INTRODUCTION

The International Prostate Symptom Score (IPSS) is used to assess lower urinary tract symptoms (LUTS) in men with bladder outflow obstruction (BOO), which is most often due to benign prostatic hyperplasia (BPH), prostate cancer or urethral stricture (1–3). The IPSS was designed to be self-administered by the patient, with speed and ease in mind, so that it can be used not only in Urology clinics, but also in a primary healthcare setting (i.e. by general practitioners) for the assessment of LUTS. Additionally, the IPSS can be performed multiple times to evaluate changes in symptom severity over months or years (4). The IPSS is an attempt to translate symptoms (which are subjective) into objective parameters (numbers). However, because of inter-individual differences in perceptions and interpretation of subjective symptoms, it is problematic to compare patients with one another in terms of symptom scores. Nonetheless, the IPSS is generally used to categorize patients into groups with minimal, moderate or severe urinary symptoms. The real value of the IPSS is in longitudinal follow-up, where changes in the individual's symptom score can be used to assess response to treatment (5). Patients with lower educational levels experience greater difficulty completing the AUA-SI or IPSS (6). Because the IPSS questions may be difficult to understand, even for men with a relatively high level of education, patients often ask the doctor or nurse for an explanation of the questions while completing the form. This invariably introduces the risk of influencing the patient's responses (7).

**Figure 1:**
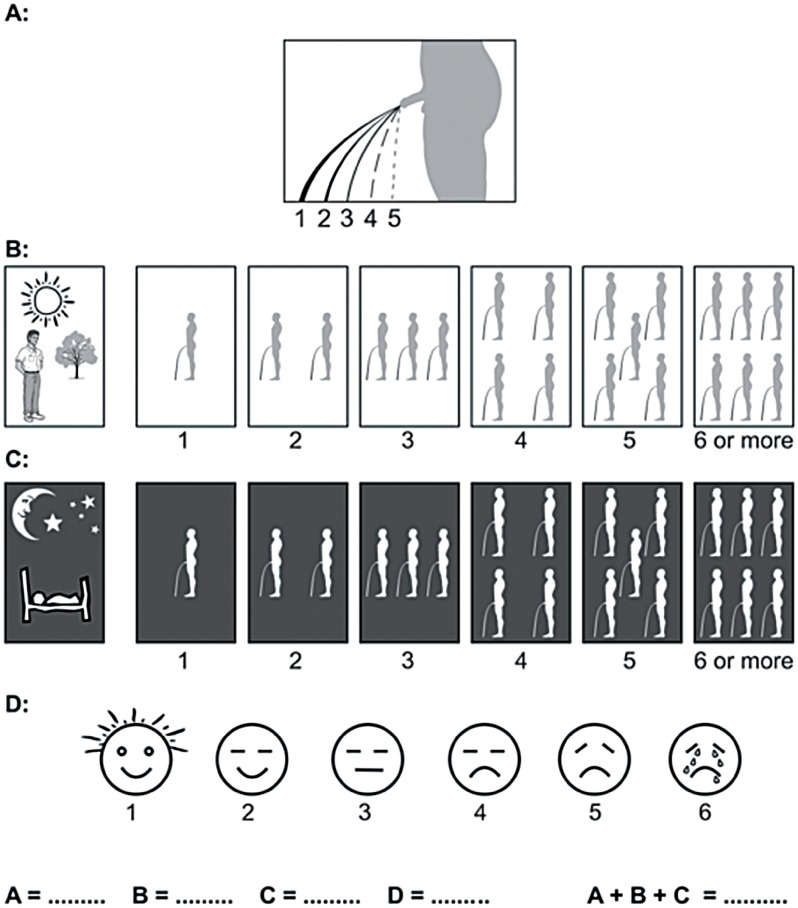
Visual prostate symptom score (VPSS) consisting of pictograms to evaluate (A) force of the urinary stream, (B) daytime frequency, (C) nocturia, and (D) quality of life.

Adam E. Groeneveld, a urologist who has worked for many years in African countries, developed a simplified assessment of the force of the urinary stream. Using this concept, we developed a Visual Prostate Symptom Score (VPSS) which also assesses urinary frequency during the day and night, and the patient's overall quality of life (Figure-1) (©Stellenbosch University).

Previous reports from our institution on the VPSS indicated a significant correlation with the IPSS score and also with maximum- and average urinary flow rates. It was also shown that men with limited education could complete the VPSS questionnaire without any assistance (7, 8). In this study, we wanted to establish if the available data on the VPSS can be verified and, if so, expand on that data.

Transrectal ultrasound (TRUS) guided prostate biopsy is the gold standard in diagnosing prostate carcinoma (9). Although the exact number of prostate biopsies performed per year is unknown, it is estimated that physicians in the United States perform about 500.000 TRUS-guided biopsies of the prostate per year (10). Previously, it was believed that the procedure is without pain or only mildly uncomfortable and as a result, most TRUS biopsies of the prostate are performed without any form of analgesia or anesthesia (9). However, a survey in the USA showed that approximately 50% of TRUS biopsies are performed with some sort of analgesia (11). Studies have consistently indicated that a number of men (about 24% to 30%) will experience significant pain during TRUS biopsies (10, 12, 13). Support for this observation was provided by Gustafsson et al. who assessed the psycho-physiological reaction to the process of prostate cancer screening (14). Psychological stress experienced by men undergoing TRUS-guided prostate biopsy may be attributable to fear of the potential diagnosis of cancer, the anal route of penetration, the fact that the examined organ is part of the male sexual system and anticipated pain (14).

Although several studies have been performed to document pain during- and after transrectal prostate biopsy using different numbers of biopsy cores and different forms of local anesthetic or analgesia, to our knowledge no prospective study has been performed to assess patient perceptions and expectations of transrectal prostate biopsy prior to and after the procedure.

## MATERIALS AND METHODS

After obtaining institutional ethics committee approval, men that presented with LUTS and who had an indication to undergo TRUS-guided prostate biopsy were included.

All were given both the IPSS, and subsequently, the VPSS questionnaire to complete and it was noted whether the patient completed the questionnaire on his own or whether he asked for assistance. In addition, the time taken (in seconds) to complete the questionnaire was also noted. Information on the patient's level of education, literacy, occupation and income were documented. Peak (Qmax), and average (Qave) urinary flow rates, voided volume, and ultrasound-measured postvoid residual urine volume were obtained for each patient.

**Figure 2:**
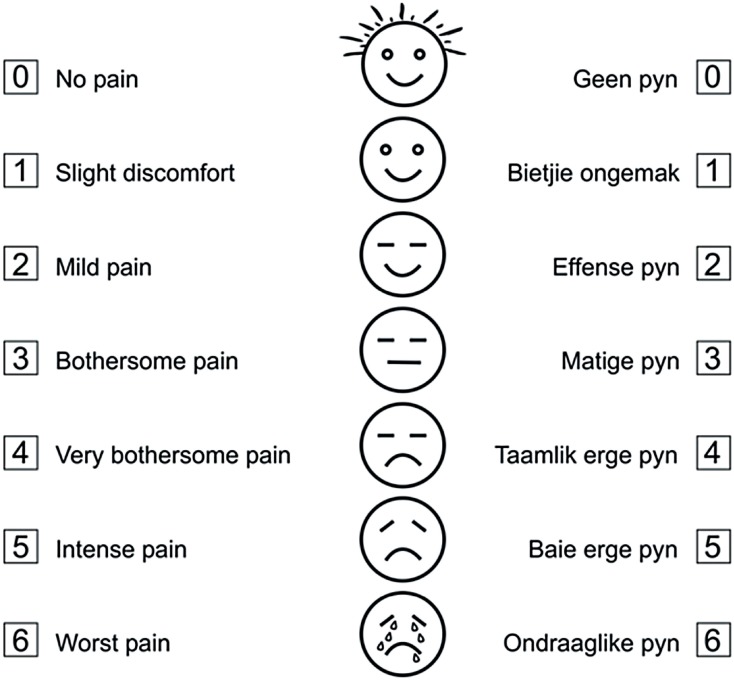
TRUS guided prostate biopsy assessment questionnaire.

The equipment necessary for the TRUS-guided prostate biopsy was shown to each patient and following this, the patient was asked to complete the TRUS-guided prostate biopsy assessment questionnaire (Figure-2), with a score of 0-6, to assess the pain or discomfort expected by him during the procedure. Immediately after completion of the biopsy procedure, the patient was asked to indicate the level of discomfort actually experienced.

An ultrasound machine with a transrectal intracorporeal probe was used for the ultrasound biopsy guidance. A semi-automatic spring coil device with an 18-gauge needle was used to obtain 8 core biopsies in all patients. TRUS was also used to calculate the prostate volume. Lubricating jelly containing lignocaine was inserted rectally in all patients before TRUS was initiated. The procedure was performed by four different providers.

Statistical analysis was performed using GraphPadInstat® software, with Student's t-test for parametric variables, Mann-Whitney test for nonparametric data, Fisher's exact test for contingency tables and Spearman's rank test for correlation analysis. A two-tailed P-value < 0.05 was accepted as statistically significant.

## RESULTS

In the period May 2010 to October 2013 a total of 100 men were evaluated (mean age 65.1, range 41-85 years). The patient's years of formal schooling were as follows: 1-7 years in 31 patients and 8-12 years in 66 patients, four had a tertiary education and three had no formal schooling.

There was a statistically significant positive correlation between the total VPSS score and total IPSS score and also between the prostate volume measured by TRUS and by digital rectal examination (DRE) (Table-1). The VPSS took significantly less time to complete than the IPSS. This was especially true for the patient groups with education grade ≤ 7 compared with grade ≥ 10. The mean time to complete the IPSS was 165 seconds (range 70-295 seconds) and 100 seconds for the VPSS (range 35-195 seconds) (p-value < 0.001). In patients with ≤ 7 education grade mean time to complete IPSS was 185 seconds and 103 seconds for the VPSS (p-value < 0.001). In patients with education grade ≥ 10 the mean time to complete IPSS was 142 seconds and 90 seconds for the VPSS (p-value = 0.038).

**Table 1:** Correlations between IPSS, VPSS, uroflowmetry parameters, and prostate volume.

	Correlation Coefficient (r)	p-value
IPSS total vs. VPSS total	+ 0.802	< 0.0001
IPSS total vs. Qmax	- 0.308	0.002
IPSS total vs. Qave	- 0.310	0.002
IPSS vs. TRUS volume	+ 0.083	0.4 (ns)
VPSS vs. Qmax	- 0.311	0.002
VPSS vs. Qave	- 0.344	< 0.001
VPSS vs. TRUS volume	+ 0.194	0.054 (ns)
Qmax vs. TRUS volume	- 0.260	0.009
Qave vs. TRUS Volume	- 0.348	< 0.001
DRE volume vs. TRUS volume	+ 0.665	< 0.001

In the group of education grade ≤ 7, only 17.6% could complete the IPSS without assistance, whereas 38.2% could complete the VPSS without assistance. This difference was not statistically significant for the education grade ≥ 10.

The mean duration of procedure for prostate biopsy was 5.7 minutes (range 2-12 min). The mean time between inserting lignocaine jelly to inserting TRUS probe was 3.6 mins (2-5 min). The mean pain score before biopsy (anticipated pain) was higher than after biopsy (experienced pain). The mean pain score before biopsy was 2.8 (range 0-6) and mean pain score after biopsy was 1.8 (range 0-5). The pain during biopsy was less than expected in 67% of patients, with the score 1 point less in 33%, 2 points less in 17%, and ≥ 3 points less in 17%. In 23% of patients the anticipated pain-and experienced pain scores were the same and in 10% of patients the experienced pain score was higher than the anticipated pain score. In the group of patients with < 7 years compared to the group with > 8 years of schooling, the mean pain score before the biopsy was significantly higher, but after the biopsy there was no significant difference. The mean pain score before biopsy for patients with < 7 years was 3.5 (range 1-6) and after biopsy was 1.6 (range 0-4). Whereas with patients > 8 years of schooling the mean score before biopsy was 2.7 (range 0-5) and then 1.8 (range 0-5) after biopsy.

The mean prostate volume estimation on DRE was 41.4 cc (25-100) and on TRUS 47.3 cc (12-148). The histological diagnoses of the prostate biopsies were as follows: adenocarcinoma in 50 patients, BPH or normal prostatic tissue in 40 and prostatitis in 10. Patients with benign rectal exam vs. malignant rectal exam had the following pain assessment scores: Mean score before biopsy 2.8 vs. 3.0 and after biopsy 1.8 vs. 1.6 (not significant). Patients with < 50 cc TRUS prostate volumes versus > 50 cc prostate volumes had the following scores: Mean score before biopsy 2.8 vs. 2.9 and after biopsy 1.8 vs. 1.7 (not significant).

## DISCUSSION

A sixth grade reading level is considered necessary to understand the questions asked in the IPSS (15). This would mean that 31% of patients in the present study would not be able to complete the IPSS without assistance. As was proven with this study, the VPSS has the advantage of being understood by men with low levels of education or even those who are completely illiterate. Two previous studies also demonstrated that the VPSS correlated positively with the IPSS and uroflowmetry parameters (7, 8). We once again confirmed that the VPSS took significantly less time to complete than the IPSS - this finding is consistent with a previous study done at our institution (8) by Wessels et al. on a different patient population. This difference in time taken to complete the questionnaire is especially relevant in those patients with a low level of education. While the IPSS is a critically important tool for assessing LUTS, patients with low educational levels have been found to report higher scores, possibly predisposing them to inappropriate care (6, 15, 16). Moreover, a visual symptom score might make translating the IPSS, originally written in English, redundant.

The VPSS has not been disseminated to, or studied at other institutions. Further multi-institutional investigation and investigation on patients with other racial or ethnic backgrounds could be considered.

TRUS-guided prostate biopsy is a common procedure, performed on an outpatient basis, to diagnose prostate cancer. Important (and possibly controversial) questions that remain unanswered, are whether patient's perceptions of pain or discomfort during TRUS-guided prostate biopsy, as well as after the procedure, warrant that the procedure be performed under general- rather than local- or no anesthesia, and also whether these perceptions of pain and discomfort relating to the diagnostic procedure may present a barrier to the early detection of prostate cancer. There are conflicting opinions regarding the question of intrarectal lidocaine (lignocaine) gel being more effective than lubricating jelly only (17, 18) in decreasing pain during TRUS biopsies of the prostate. The protocol in our Urology Department is to use intrarectal lignocaine gel routinely.

The reason for using a TRUS-guided prostate biopsy assessment questionnaire with score ranging from 0-6 (Figure-2) and not a more standardized score like the Visual Analog Scale with scores ranging from 0-10, was to make the interpretation simpler by matching a number with text with a picture at each score.

Previous investigations have shown that even when anesthesia-free, TRUS-guided prostate biopsy was considered to be only mildly uncomfortable for most patients, 19% judged that it should be accompanied by some type of anesthesia and 6% of patients judged that the procedure was so uncomfortable that it should have been performed under general anesthesia (19).

Understanding the expectations and reactions of men having to undergo TRUS-guided prostate biopsy could be extremely useful. In this study we have shown that, for our population, the perceptions of men with regard to discomfort or pain during TRUS-guided prostate biopsy are more negative prior to undergoing the examination than the actual pain experienced as reported after the biopsy.

With this finding and the fact that overall post-biopsy pain scores were low, it is reasonable to assume that our practice of prostate biopsy using lidocaine gel only is acceptable to our patients. When counseling a patient before prostate biopsy, the patient can be reassured, because the pain experienced will most likely be less than anticipated.

A possible consideration for why the post-biopsy scores were so low is the fact that eight core biopsies were taken in all patients, and not more extensive numbers or saturation biopsies.

Although there is no consensus on this, discomfort during transrectal biopsy does appear to be proportional to the number of cores taken (20). However, Mariappan et al. demonstrated that increasing the number of cores did not increase pain scores (21). Seeing that it is more common practice to perform 10-12 cores with the baseline biopsy, it could be postulated that the pain scores in this study can't be generalized to patients receiving more core biopsies and this is a limitation of the study.

Patients with less than seven years of schooling had a significantly higher score before the biopsy than those with more than eight years of schooling. Although the exact reasons for this are not clear, a possible explanation may be that educated patients were more likely to read (online or patient brochures) about the procedure beforehand and that this may have ensured more realistic expectations of pain to be experienced during the procedure.

Showing the biopsy equipment to the patients prior to them completing the score questionnaire may have altered the pre-biopsy scores-this is a possible limitation of the study method.

Another possible limitation of the study is that the procedure was performed by four different providers. Some providers might have had more experience with TRUS-guided biopsy and performed a more rapid, and possibly less uncomfortable, procedure which could lead to different results.

## CONCLUSIONS

In men with LUTS scheduled to undergo prostate biopsy, there was a statistically significant positive correlation between the total VPSS- and total IPSS scores. In men with a limited level of education, the VPSS took significantly less time to complete than the IPSS.

Patient's perception of expected pain or discomfort during TRUS- guided prostate biopsy was significantly higher than the pain actually experienced during biopsy. Men with lower education levels had significantly higher expectation of pain prior to biopsy, but reported similar mean pain score during biopsy when compared to men with higher education level. There was no significant difference in pain experienced by patients with malignant prostates compared to benign prostates or with larger- compared to smaller prostates.

## References

[1] Wadie BS, Badawi AM, Ghoneim MA (2001). The relationship of the International Prostate Symptom Score and objective parameters for diagnosing bladder outlet obstruction. Part II: the potential usefulness of artificial neural networks. J Urol..

[2] Bosch JL, Hop WC, Kirkels WJ, Schröder FH (1995). The International Prostate Symptom Score in a community-based sample of men between 55 and 74 years of age: prevalence and correlation of symptoms with age, prostate volume, flow rate and residual urine volume. Br J Urol..

[3] Eckhardt MD, van Venrooij GE, Boon TA (2001). Symptoms and quality of life versus age, prostate volume, and urodynamic parameters in 565 strictly selected men with lower urinary tract symptoms suggestive of benign prostatic hyperplasia. Urology..

[4] Rodrigues P, Meller A, Campagnari JC, Alcântara D, D’Império M (2004). International Prostate Symptom Score--IPSS-AUA as discriminat scale in 400 male patients with lower urinary tract symptoms (LUTS). Int Braz J Urol..

[5] Lukacs B, Grange JC, Comet D (2000). One-year follow-up of 2829 patients with moderate to severe lower urinary tract symptoms treated with alfuzosin in general practice according to IPSS and a health-related quality-of-life questionnaire. BPM Group in General Practice. Urology..

[6] Johnson TV, Abbasi A, Ehrlich SS, Kleris RS, Schoenberg ED, Owen-Smith A (2008). Patient misunderstanding of the individual questions of the American Urological Association symptom score. J Urol..

[7] van der Walt CL, Heyns CF, Groeneveld AE, Edlin RS, van Vuuren SP (2011). Prospective comparison of a new visual prostate symptom score versus the international prostate symptom score in men with lower urinary tract symptoms. Urology..

[8] Wessels SG, Heyns CF (2014). Prospective evaluation of a new visual prostate symptom score, the international prostate symptom score, and uroflowmetry in men with urethral stricture disease. Urology..

[9] Hollingsworth JM, Miller DC, Wei JT (2006). Local anesthesia in transrectal prostate biopsy. Urology..

[10] Moinzadeh A, Mourtzinos A, Triaca V, Hamawy KJ (2003). A randomized double-blind prospective study evaluating patient tolerance of transrectal ultrasound-guided biopsy of the prostate using prebiopsy rofecoxib. Urology..

[11] Davis M, Sofer M, Kim SS, Soloway MS (2002). The procedure of transrectal ultrasound guided biopsy of the prostate: a survey of patient preparation and biopsy technique. J Urol..

[12] Zisman A, Leibovici D, Kleinmann J, Siegel YI, Lindner A (2001). The impact of prostate biopsy on patient well-being: a prospective study of pain, anxiety and erectile dysfunction. J Urol..

[13] Peyromaure M, Ravery V, Messas A, Toublanc M, Boccon-Gibod L, Boccon-Gibod L (2002). Pain and morbidity of an extensive prostate 10-biopsy protocol: a prospective study in 289 patients. J Urol..

[14] Gustafsson O, Theorell T, Norming U, Perski A, Ohström M, Nyman CR (1995). Psychological reactions in men screened for prostate cancer. Br J Urol..

[15] MacDiarmid SA, Goodson TC, Holmes TM, Martin PR, Doyle RB (1998). An assessment of the comprehension of the American Urological Association Symptom Index. J Urol..

[16] Netto NR, de Lima ML (1995). The influence of patient education level on the International Prostatic Symptom Score. J Urol..

[17] Chang SS, Alberts G, Wells N, Smith JA, Cookson MS (2001). Intrarectal lidocaine during transrectal prostate biopsy: results of a prospective double-blind randomized trial. J Urol..

[18] Issa MM, Bux S, Chun T, Petros JA, Labadia AJ, Anastasia K (2000). A randomized prospective trial of intrarectal lidocaine for pain control during transrectal prostate biopsy: the Emory University experience. J Urol..

[19] Irani J, Fournier F, Bon D, Gremmo E, Doré B, Aubert J (1997). Patient tolerance of transrectal ultrasound-guided biopsy of the prostate. Br J Urol..

[20] Autorino R, De Sio M, Di Lorenzo G, Damiano R, Perdonà S, Cindolo L (2005). How to decrease pain during transrectal ultrasound guided prostate biopsy: a look at the literature. J Urol..

[21] Mariappan P, Chong WL, Sundram M, Mohamed SR (2004). Increasing prostate biopsy cores based on volume vs the sextant biopsy: a prospective randomized controlled clinical study on cancer detection rates and morbidity. BJU Int..

